# A collaborative approach to develop an intervention to strengthen health visitors’ role in prevention of excess weight gain in children

**DOI:** 10.1186/s12889-022-14092-x

**Published:** 2022-09-13

**Authors:** Devashish Ray, Falko Sniehotta, Elaine McColl, Louisa Ells, Gill O’Neill, Karen McCabe

**Affiliations:** 1 Population Health Sciences Institute, Newcastle University, Newcastle-upon-Tyne, NE2 4AX UK; 2 School of Clinical and Applied Sciences, Leeds Beckett University, Leeds, England; 3 Department of Public Health, Durham County Council, Durham, England

**Keywords:** Intervention development, Behaviour change, Guideline implementation, Childhood obesity, Health visitors

## Abstract

**Background:**

The high prevalence of childhood obesity is a concern for public health policy and practitioners, leading to a focus on early prevention. UK health visitors (HVs) are well-positioned to prevent excessive weight gain trends in pre-school children but experience barriers to implementing guideline recommended practices. This research engaged with HVs to design an intervention to strengthen their role in prevention of early childhood obesity.

**Methods:**

We describe the processes we used to develop a behaviour change intervention and measures to test its feasibility. We conducted a systematic review to identify factors associated with implementation of practices recommended for prevention of early childhood obesity. We carried out interactive workshops with HVs who deliver health visiting services in County Durham, England. Workshop format was informed by the behaviour change wheel framework for developing theory-based interventions and incorporated systematic review evidence. As intended recipients of the intervention, HVs provided their views of what is important and acceptable in the local context. The findings of the workshops were combined in an iterative process to inform the four steps of the Implementation Intervention development framework that was adapted as a practical guide for the development process.

**Results:**

Theoretical analysis of the workshop findings revealed HVs’ capabilities, opportunities and motivations related to prevention of excess weight in 0-2 year olds. Intervention strategies deemed most likely to support implementation (enablement, education, training, modelling, persuasion) were combined to design an interactive training intervention. Measures to test acceptability, feasibility, and fidelity of delivery of the proposed intervention were identified.

**Conclusions:**

An interactive training intervention has been designed, informed by theory, evidence, and expert knowledge of HVs, in an area of health promotion that is currently evolving. This research addresses an important evidence-practice gap in prevention of childhood obesity. The use of a systematic approach to the development process, identification of intervention contents and their hypothesised mechanisms of action provides an opportunity for this research to contribute to the body of literature on designing of implementation interventions using a collaborative approach. Future research should be directed to evaluate the acceptability and feasibility of the intervention.

**Supplementary Information:**

The online version contains supplementary material available at 10.1186/s12889-022-14092-x.

## Background

Childhood obesity is an urgent global public health concern. Improved understanding of maternal and infant risk factors has put emphasis on the role of primary care practitioners (PCPs) in prevention of excess weight gain during the first 2 years of life [1]. In England and the rest of UK, health visitors (nurses or midwives with additional training in public health nursing) who lead the delivery of the Healthy Child programme (HCP) 0-5 have a key role in promoting healthy weight gain in pre-school children [2]. During the mandated visits within the HCP 0-5, HVs are expected to monitor the infant’s health, nutrition, and growth, assess risk of excessive weight gain, and provide consistent, evidence-based messages on nutrition, managing weight gain and physical activity [3]. HVs are encouraged to use every opportunity to discuss the importance of a healthy weight and lifestyle with parents, and signpost to relevant national resources and to relevant local community activities [4].

Trained practitioner-led family-based childhood obesity prevention programmes hold promise [5]. A programme called HENRY (Health, Exercise, Nutrition in the Really Young) that is reported to be currently commissioned by 40 local authorities across the UK [6] and delivered by HVs and early years staff has demonstrated the potential of targeting parents as agents of change, not only to establish healthy weight trajectories in the child but also to support positive parenting practices, and to influence healthy weight behaviours for the family [7]. However, PCPs including HVs do not consistently implement guideline recommended practices. Studies show that many PCPs do not routinely use the BMI chart but rely instead on simple visual inspection to assess child’s weight status [8, 9], do not routinely discuss and provide breastfeeding advice during antenatal and postnatal visits [10, 11], and less frequently discuss healthy eating and physical activity with parents of 0-2 year olds as compared to parents of school aged children [12–14]. PCPs, including HVs have described lack of skills and confidence in engaging with parents to discuss weight related topics, especially if they lacked relevant training and resources, and if parents have excess weight and/or are perceived as not motivated [15]. Training which provides opportunities for skills development, encourages reflection on practice, and draws PCPs’ attention to differences between current practice and desired standards has the potential to improve outcomes for PCPs (professional development) [16] and children and families [17].

Interventions designed to change practice behaviours and improve the uptake of guidelines are invariably complex as they usually require an integrated set of actions and processes to address specific barriers. The Medical Research Council (MRC) recommends using best available evidence and appropriate theory (to understand the likely pathway(s) of behaviour change and how change is to be achieved) for intervention development [18]. The Behaviour Change Wheel (BCW) framework [19], developed by synthesis of 19 theoretical frameworks of behaviour change, provides a systematic approach to incorporate theory into the intervention development process and complements the MRC framework for the development of complex interventions. At the hub of the BCW is the Capability, Opportunity, Motivation-Behaviour (COM-B) model, an aggregated theoretical model of behaviour which can be used to conduct an analysis of the target behaviours. The COM-B postulates that the interactions between an individual’s capability (C), opportunity (O) and motivation (M) provide explanations about why a behaviour (B) is or is not performed. The components of the COM-B model can be further elaborated using the Theoretical Domains Framework (TDF), an integrated framework comprising 14 psychological domains that are hypothesised to influence behaviour [20]. The BCW framework includes nine intervention functions, seven policy categories, and links to a taxonomy of 93 behaviour change techniques (BCTs) which are suitable for developing intervention options and content, following the COM-B behavioural analysis. The BCW has been applied across different topics, target groups and organisational contexts to design complex interventions [21–23].

Interventions developed through a collaborative approach between researchers and stakeholders are regarded as more likely to be feasible to deliver, to maximise uptake of the intervention, and to facilitate the process of translating research evidence into practice [24]. One collaborative approach is co-design where expertise and experiences of stakeholders contribute to intervention design. Collaborative approaches between researchers and healthcare professionals have been successfully demonstrated in the designing of interventions in primary care [22, 25].

This paper describes the systematic development of an intervention in which stakeholder engagement was combined with the steps of the BCW framework and an evidence-based approach. The aim of this research was to develop an intervention to strengthen HVs’ role in prevention of excess weight gain in 0-2 year olds.

### Research setting and participants

The study which formed part of a doctoral research project, was suggested and co-funded by Durham County Council (DCC) public health department to support professional practice development of HVs who deliver the HCP 0-5 across areas within County Durham. During the time this research was undertaken (2019), the HCP 0-5 was delivered in the County by the Growing Healthy Team, Harrogate, and District NHS Foundation Trust (HDFT). County Durham is a large predominantly rural area home to around 530,000 people (2019 estimates) in Northeast England; children aged 0-4 years constitute around 6% of the population [26]. County Durham has significant health and social problems related to economic deprivation. In 2018/19, the prevalence of excess weight in children aged 4-5 year in County Durham (25%) was significantly higher than the average for England (23%), with significant socioeconomic disparities within different areas of the County [27]. Further, the prevalence of several modifiable risk factors for childhood obesity is higher (or worse) in the County than the national average [28]. The County’s Healthy Weight Alliance has identified “best start in life” which focuses on the health of 0–2-year-olds as one of several work streams for implementation of a whole systems approach to obesity prevention [29].

HVs and their supervisors (as the stakeholder group) were involved as research participants in this study. Five HV teams were identified who worked across different rural and urban areas within County Durham. In February 2019, there were a total of 128 HVs (equivalent to 106.6 whole time equivalent staff) in post across the County, with the number of HVs per team ranging between 21 and 32.

Ethical approval was granted by Health and Care Research Wales (19/HRA/0920) in February 2019. HDFT which employed the HVs who participated in this study granted permission to conduct the study.

## Development of the intervention

**Fig. 1:**
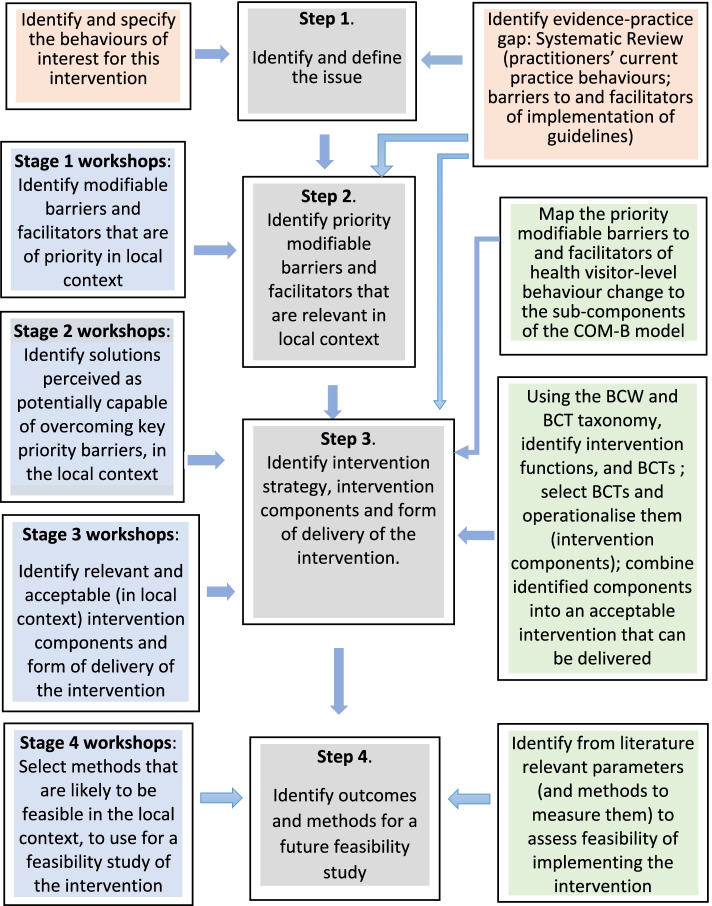
An overview of the development of the intervention. Boxes shaded grey represent the four steps of the Implementation Intervention framework; boxes shaded pink represent activities undertaken prior to the co-design workshops; boxes shaded blue represent the stages of the workshop with HVs; boxes shaded green represent desktop research activities; BCW = Behaviour Change Wheel; BCT = Behaviour change technique; COM-B = Capability, Opportunity, Motivation- Behaviour model

The intervention development process involved a series of steps, as shown in Fig. 1, and was guided by adapting the four-stepped approach outlined in the Implementation Intervention development framework [30]. This framework provides a systematic method for developing a theory-based intervention to change practice behaviours and has been used to guide the development of implementation interventions in diverse healthcare settings [31–34]. The four steps were: (1) identify and define the issue; (2) identify what barriers and facilitators need to be addressed; (3) identify intervention strategy, intervention components and form of delivery; (4) identify outcomes and methods for a future feasibility study of the intervention. A collaborative approach was used to co-design the intervention with HVs as professional stakeholders. As illustrated in Fig. 1 above, this collaborative work involved four stages of workshops, to meet the objectives of steps 2, 3, and 4 of the intervention development process.

### Stakeholder engagement process

Prior to seeking approvals for this project, the lead researcher (DR) consulted with health visiting service managers and all five HV teams and presented an overview of the research project, including the anticipated role of HVs as end-users of the intervention. Purposive sampling of teams with respect to which team participated in which workshop was used to ensure representativeness of the views and experiences of the HVs who worked in different areas within the county.

Eleven workshops (three in Stage one, two in Stage two, three in Stage three, and three in Stage four) were conducted. The workshops lasted between 60 and 75 minutes. The decision about the number of workshops conducted at each of the four stages was informed by the nature of data generated from each workshop. The workshops were held at venues across the local authority area where HVs hold routine monthly staff meetings and followed on immediately after those meetings. The scheduling of dates and time slots for the workshops and the choice of workshop location ensured members of all the five HV teams had the opportunity to take part in a minimum of two workshops. The number of participants in each workshop was determined by the size of the HV team which took part in that workshop. Table 1 shows the participating HV teams and the number of participants at each workshop.

**Table 1:** Participating health visiting teams and number of participants at the workshops

Stage of the workshops	Number of workshops within each stage	Participating health visiting teams; Number (n) of participants (HVs) at each workshop (WS)
1	Three	WS 1 (team A), *n* = 18; WS 2 (team B), *n* = 11; WS 3 (team C), *n* = 24
2	Two	WS 4 (team D), *n* = 20; WS 5 (team C), *n* = 14
3	Three	WS 6 (team A), *n* = 10; WS 7 (team E), *n* = 6; WS 8 (team D), *n* = 10
4	Three	WS 9 (team C), *n* = 20; WS 10 (team E), *n* = 8; WS 11 (team B), *n* = 6

HVs were engaged in the ‘informed’ mode of co-design [35] where in a consultative role, they provided their views of the contextual relevance, feasibility, and acceptability of the emerging intervention. The workshops were conducted between May and October 2019. The overall planning, facilitation and evaluation of the workshops were informed by values and design principles recommended for stakeholder engagement in research [36]. All workshops were facilitated by DR. An experienced specialist public health nurse took on the role of the co-facilitator. Co-production principles [37] informing the workshops included: (1) creation of an environment that is safe for everyone to participate, (2) a structured approach where participants are actively engaged to contribute, and (3) a process where participants’ opinions are heard, evaluated, and acted upon. A pre-designed questionnaire (an open question was included to enable HVs to elaborate on their responses) was used to gather feedback from workshop participants about their experiences of participation. The planning of workshop-specific activities was informed by the objectives of that particular workshop and consideration of issues such as the time and resources available at the venue and expected number of participants. Table 2 (page 11) presents an overview of the stages of the workshops, their aims, activities, and related post-workshop activities. Pre-prepared topic guides were used to guide the activities that were carried out during each stage of the workshops. Participants were provided with activity sheets (instructions) and a written summary of the outputs of the previous workshops where applicable. Both quantitative (dot voting for ranking activities) [38] and qualitative methods (group discussions, brain storming, post-it notes exercises) [39] were concurrently used to collect information from participants.

**Table 2:** An overview of the stages of the workshops and post-workshop activities undertaken for the co-designing of the intervention

Workshop stages	Workshop (WS) activities	Post workshop activities
**Stage 1** Identify priority and potentially modifiable barriers and facilitators	WS 1 and 2 • Spontaneously identify barriers and facilitators of practices that are relevant in the local context • Assess relevance in the local context of 20 barriers and 10 facilitators that were identified as key findings in a recently completed SR	• Identify barriers that were common to the SR and participants, barriers unique to the SR, and barriers unique to participants • Identify 20 key barriers from the analyses: *this list was used as an input for Stage 1 WS 3*
	WS 3 • Rating of key barriers (*n* = 20) in terms of their importance and changeability in the local context • Identify training and resource needs	• Priority ranking of key barriers: *used as an input for the next stage (stage 2) of workshops* • Prepare summary of priority training and resource needs: *used as an input for stage 2 workshops*
**Stage 2** Identify potential solutions	• Identify ideas for interventions considered by participants as potentially helpful • Categorise proposed ideas for interventions in terms of the target recipient group: HV, parent and service provider organisation	• Select suitable intervention strategy • Theoretical analysis of HV-level barriers and facilitators • Identify relevant intervention functions and potentially useful BCTs; operationalise the BCTs; operationalised BCTs were *used as inputs for stage 3 workshops*
**Stage 3** Select BCTs and their mode of delivery	• Rate potentially relevant BCTs for their importance and acceptability in local context • Identify HVs’ perspectives of (1) relevant topics and activities for an interactive training intervention; and (2) factors that can facilitate/ promote HVs’ participation and enhance their experience of participation	• Select BCTs (and their modes of delivery) assessed as important and acceptable in the local context by participants; combine the selected BCTs into a cohesive, deliverable intervention • Develop the draft of an interactive face-to-face training intervention
**Stage 4** Select feasibility outcomes and methods	• Rate the importance of parameters and the feasibility of the methods to estimate them (they were identified from relevant literature), in the local context	• Select feasibility outcomes (parameters) and methods that could be used for a feasibility study of the intervention

*WS* workshop, *SR* systematic review, *BCT* behaviour change technique

### Approach to data analysis

The workshop activities generated diverse types of data. These data represented participants’ decisions about contextual relevance, priority ranking and rating for acceptability/importance of items; ideas about intervention content; and preliminary analytical work carried out by participants of self-generated data from workshop activities. Data analysis was an iterative and ongoing process. Qualitative data were analysed using the Framework Analysis method [40]. Descriptive statistics were used to summarise the numerical data generated from various dot voting activities. Where appropriate, the analysis of the quantitative data representing rating of relevance (or non-relevance) of items, acceptability, and feasibility (in the local context) were triangulated with the concepts and themes identified from the analysis of the qualitative data, to establish corroboration of the evidence from the two sets of data [41]. The results from the analyses were grouped together into “findings” to inform the specific stages of development of the intervention.

Because of the iterative nature of this work, the development of the intervention is reported step by step, including the objectives, methods, and findings relevant for that step.

## Step 1: identify and define the issue

The work completed in this step laid the groundwork for the designing of the intervention.

### Identify and specify the behaviours

#### Method

The behaviours were identified from the HCP 0-5 framework for action [42], guidelines published by UK’s National Institute for Health and Care Excellence [43–46] and by Public Health England [2, 4]. The behaviours were specified using the AACTT (Action, Actor, Context, Target, Time) framework [47] by asking the questions: what is the clinical behaviour (or series of linked behaviours) (Action); who performs the behaviour(s) (Actor – this could be an individual practitioner or a team); when (Time) and where (Context) do they perform the behaviour(s); and with whom (or for whom) the behaviour is performed (Target)?

#### Findings

A number of practice behaviours that are relevant to this research were identified. The behaviours that form part of a larger behaviour were grouped together into “behaviour areas” and specified according to the AACTT framework, as shown in Table 3. These behaviours are supported by strong evidence, are expected to be performed by the HV (or health visiting staff) during their mandated contacts with 0-2 year old children and their parents and are potentially modifiable at individual HV-level.

**Table 3:** Specification of health visitors’ practice behaviours relevant for this study

Actor	Health visitor or HCP 0-5 staff
**A**ctions	**Behaviour area**: Monitor weight and growth. Plot and record weight and height/length of the child on appropriate growth percentile charts (frequency as recommended in guidelines); interpret and assess risk of excess weight gain; discuss findings with parents
	**Behaviour area**: Assess and communicate risk of excess weight. Assess parent-level risk factors; assess infant diet and nutrition, feeding practices, physical activity, sedentary behaviours (screen time use), and sleep; communicate risk of excess weight gain to parents/carers; assess parents’ readiness and motivation to change
	**Behaviour area**: Health promotion and prevention of excess weight Provide tailored and practical advice and support; use recommended approaches to reinforce consistent health promoting messages; guidance and support for behaviour change; provide information about community programs; referrals to other practitioners and/or services when indicated by guidance
**C**ontext and **T**ime	Visits/reviews at home/health centre as specified by service provider organisation; any HV- or parent-initiated contact on topic of infant’s weight, diet and feeding practices, sleep, physical activity, and sedentary activity.
**T**arget	0-2 year old children and their parent(s)/carer(s)

### Identify the evidence-practice gap

#### Method

We conducted a mixed-methods systematic review (SR), the methods and the findings of which have been published elsewhere [48]. The review synthesised the evidence on gaps in implementation of guideline recommended practices for prevention of excess weight in children aged 0-5 years; and barriers to and facilitators of implementation, as perceived by PCPs. The barriers and facilitators were categorised into the subcomponents of the COM-B model of behaviour.

#### Findings

Our SR included 50 studies from nine countries [48]. Nurses with a specialist public health role (such as UK health visitors and their counterparts in other countries) were identified as the sole participant group in 10 studies and as one participant group in nine studies that used mixed samples. The review found that PCPs inconsistently address childhood obesity prevention. Implementation varied in terms of PCPs’ views about the importance of the practice behaviour and their beliefs about the time and the skills required in delivering them. PCPs identified several barriers which influenced their capability, opportunity, and motivation to perform the behaviours; these were insufficient knowledge of childhood obesity prevention and lack of confidence in their communication skills, concerns about risk of harm to their relationship with parents, low expectations of outcomes of prevention efforts, time constraints, and parental lack of concern/motivation to change. However, when PCPs were specifically trained to address childhood obesity in their routine practice, they were more likely to implement recommended practices. A trusting relationship between PCP and the parent was essential for PCPs to discuss weight related behaviours; whilst this potentially facilitated their practice, the value attached to maintaining the relationship acted as a barrier. The review also identified innovative communication strategies used by PCPs to overcome barriers, and resource and training needs of PCPs. The review findings indicated that embedding early-childhood obesity prevention practices into PCPs’ existing routines will require support for the practitioner’s role, such as clear care pathways, decision support tools, and access to training and referral services.

## Step 2. Identify priority barriers and facilitators that are relevant in local context

### Identify locally relevant barriers and facilitators

#### Method

Participants of stage one workshops spontaneously mentioned factors at the level of the parent/family, HVs, and the service provider organisation that they perceived as barriers to and facilitators of their practices in the local context. Subsequently, participants rated the contextual relevance of the barriers and facilitators that were identified in the recently completed SR.

#### Findings

The majority of barriers and facilitators spontaneously mentioned by participants (summarised in Tables 4 and 5) were also identified within the SR. Participants mentioned many barriers external to them, more specifically barriers at the levels of the parent and service provider. Almost all the barriers and facilitators unique to the SR (i.e., not spontaneously mentioned by participants) were rated as contextually relevant by the majority of workshop participants. A summary of the findings of rating for contextual relevance of SR-identified factors is presented in Additional files 1 (barriers) and 2 (facilitators).

**Table 4:** Barriers spontaneously mentioned by participants

Level of the barrier	Description of the barriers
Practitioner	Limited knowledge; lack of familiarity with guideline content; disagreement with guideline content; lack of confidence; concern about offending parent; harm to relationship with family
Parent (beliefs of HVs)	Socioeconomic situation; lack of understanding; lack of motivation and concern; families with complex multiple issues; misperception of healthy child weight; influence of grandparents; parental lifestyle
Organisation	Lack of practice tools; time constraints/ competing priorities; lack of united approach to the ‘problem’; lack of role support (training, resources, funding); regular weight monitoring of 0-2 year olds not a key performance indicator of HV services
Environment	Availability of baby foods in UK supermarkets marked as appropriate for 4 month old infants

**Table 5:** Facilitators spontaneously mentioned by workshop participants

Level of the facilitator	Description of the facilitators
Practitioner	Awareness of guideline content, awareness of local services, positive relationship with parent/ family
Parent (beliefs of HVs)	Receptive and engaged parents
Organisation	Collaborative working with different practitioner groups; availability of resources; support from doctors of nurses’ decisions; availability of referral services; adequate staffing (continuity of care)

### Priority ranking of the barriers

#### Method

We selected 20 barriers (and assigned them a unique identifying label) (listed in Table 6, below) out of an initial list of 23 barriers (see Additional file 1). Of these, 16 barriers were spontaneously mentioned by participants *and* also identified in the SR. The rationale for selecting the other 4 barriers is outlined in Table 6.

**Table 6:** List of the barriers (*n* = 20) selected for priority ranking

Sixteen barriers mentioned by participants *and* also identified in the SR		
Level of the barrier	Brief description (identifying label)	
Practitioner (PCP)	Lack of knowledge, skills, and confidence (P1)	
	Lack of familiarity with guideline (P2)	
	Disagreement with guideline/ evidence underpinning the guideline (P3)	
Practitioner-parent interaction	Harm to practitioner-parent relationship (P7)	
	Fear of offending parents (P8)	
Family (assumptions of PCPs)	Socioeconomic challenges (F1)	
	Lack of motivation to change (F2)	
	Families with multiple complex problems (F3)	
	Lack of understanding and skills (F5)	
	Parental excess weight and lifestyle (F6)	
	Misperception of healthy infant weight gain (F7)	
Organisation	Lack of training (O1)	
	Lack of tools and resources (O2)	
	Lack of time (O3)	
	Lack of collaboration between practitioner groups (O4)	
	Lack of role support from organisation (O5)	
Four barriers that were identified in the SR but were *not* spontaneously mentioned by participants		
Level of the barrier	Brief description (identifying label)	Rationale for including them for ranking
Practitioner	Belief: my advice does little to prevent obesity (P4)	Frequently reported as a barrier in the SR; 53% of participants rated it as locally relevant
	Uncertainty about identifying infants as being affected with excess weight (P5)	66% rated it as not locally relevant; Included because: (i) frequent finding in the SR; (ii) HVs reported very low use of BMI and uncertainty about relevance of BMI in 0-2; this makes it difficult for HVs to identify excess weight gain in infants
	Belief: primary prevention is parents’ responsibility (P6)	More than half (53%) of participants rated this barrier as relevant; 34% rated it as not relevant
Parent	Unhealthy infant/ child feeding practices (F4)	Frequently reported as a parent-level barrier in the SR; 85% of participants rated it as relevant

#### Findings

The priority ranking analysis (Additional file 3) revealed that the top four priority barriers were at the level of the individual practitioner: practitioner’s disagreement with guideline/ evidence underpinning the guideline(s); lack of knowledge, skills, and confidence; uncertainty about identifying infants as having excess weight; and lack of familiarity with guideline content. All parent-level barriers were rated high for importance but low for changeability. The list of the top 10 priority ranked barriers is presented in Additional file 4.

## Step 3. Identify intervention strategy and intervention components, and form of delivery

### Determine intervention strategy

#### Method

Participants of stage two workshops generated ideas for intervention strategies and actions that could address contextually relevant barriers at the level of the HV, parent/ family, and the service provider organisation, and create facilitators to provide role support for HVs. The findings of the stage one and stage two workshops were combined to inform the selection of the intervention strategy.

#### Findings

Participants’ ideas for interventions were categorised into workflow-focused and practitioner-focused interventions. Workflow-focused interventions sought to minimise parent- and service provider-related barriers and enhance role support for HVs. The practitioner-focused interventions sought to minimise barriers and create facilitators at individual HV-level (e.g., obesity prevention training). These findings suggested that several interventions appear to be necessary to address key barriers at the level of the individual practitioner, service provider organisation, and the parent/family. The findings are in accordance with the evidence synthesised from the SR [48] which suggested that change at multiple levels is required to produce sustainable improvement in PCPs’ adherence to guidelines.

To select the most suitable intervention strategy, we took into consideration the priorities of DCC which commissions the HV-led HCP 0-5 service at the research site, and the needs of HVs. The findings from stage one workshops revealed several modifiable HV-level barriers; importantly, the top four priority ranked barriers were HV-level barriers. The findings of the SR and practitioner-focused interventions identified by stage two workshop participants demonstrated the importance for obesity prevention training. Improving HVs’ implementation of guidelines will also require addressing key organisational-level barriers (e.g., change in service provision). Policies to support those changes (e.g., new guidelines and care pathways) will need to be implemented at the national rather than the local level [49]. These findings were discussed by members of the research team following which it was decided to develop an intervention targeting individual HV-level barriers.

### Identify what needs to change

#### Method

The HV-level barriers, including HVs’ beliefs and assumptions about parent-level factors, were mapped to the COM-B model components to identify what changes in capability, opportunity, and motivation might be needed to increase HVs’ uptake of guideline recommended practices.

#### Findings

The COM-B analysis of the barriers revealed that psychological capability, motivation (reflective and automatic), and opportunity (social and physical) are all potentially relevant drivers for HVs to perform the recommended practices. This ‘behavioural analysis’ informed what needs to happen for the target behaviours to occur and what, in terms of capability, motivation and opportunity, needs to change, as shown in Table 7.

**Table 7:** Mapping of the HV-level barriers to the domains of Capability, Opportunity, Motivation model of behaviour (COM-B)

Potentially modifiable barriers	Relevant COM-B components	What needs to happen at individual HV-level, for the target behaviours to occur
• Lack of knowledge, skills, and confidence • Lack of familiarity with guidelines/ guideline content	Psychological capability	• Understanding of the causes and consequences of rapid weight gain during infancy • Having the knowledge and skills to tailor interventions and device strategies when required • Having the confidence that they can perform the recommended practices even when experiencing parental resistance/ lack of interest
• Uncertainty about identifying infants as having excess weight • Belief: Disagreement with guidelines/evidence • Belief: my advice does little to prevent childhood obesity	Reflective motivation	• Understanding of the consequences of delay in intervention to prevent rapid infant weight gain • Having knowledge of the quality and strength of evidence underpinning guideline recommendation • Believing that HVs’ preventive efforts have the potential to produce positive health outcomes for the child and family
• Belief: preventing excess weight gain in young children is primarily parents’ responsibility • Belief: parents lack motivation to change • Belief: Parents lack knowledge and parenting skills • Parents misperceive heavier infants as healthier • Belief: Harm to practitioner-parent relationship • HVs lack time and have many competing priorities to manage during their visits • HVs lack tools and resources	Reflective motivation Social Opportunity Physical opportunity	• Believing that motivating a parent who appears to be not concerned is part of their role • Believing that providing parents with information, advice and support can help improve parents’ skills and confidence • Believing that correcting parents’ misperceptions of healthy infant weight gain is part of their role • Have the skills to manage parental resistance (actual or perceived) and sensitively engage with parents • Believing that even if resistance is experienced, discussing the topic will influence the perception of parents (and potentially their practices) • HVs having the skills and confidence to provide advice in a manner that does not threaten their existing relationship with the families • HVs prioritising discussing weight related behaviours especially when assessment suggests increased risk of rapid infant weight gain • Having skills and tools (e.g., decision making, guideline summaries, prompts) to perform the behaviours quickly and efficiently
• Sensitive topic: fear of offending parents/provoking negative reactions and emotions from parents	Automatic motivation Social opportunity	• Adopting the position that development of excess weight is a societal and environmental issue, whilst at the same time emphasising the importance of implementing practices that are known to promote healthy infant weight and prevent excessive weight gain • Feeling the need to change some existing practice routines: able to resist the instinct to avoid the topic (not wanting to ‘rock the boat’) • Recognising that it can be difficult for parents to initiate the topic because of the social stigma associated with obesity

### Identify intervention functions

#### Method

The guidance from the BCW along with the APEASE criteria [19] informed the selection of intervention functions to address the factors identified in the COM-B behavioural analysis. Applying the APEASE criteria enabled the selection of context-specific intervention functions based on **a**ffordability, **p**racticability, **e**ffectiveness/ cost-effectiveness, **a**cceptability, **s**ide-effects/safety, and **e**quity.

#### Findings

Five intervention functions were assessed as potentially capable of addressing the changes required at individual HV-level and meet the APEASE criteria. They were Education, Training, Persuasion, Modelling and Enablement. The APEASE criteria ratings for the different intervention functions are presented in Additional file 5.

### Identify intervention components

#### Method

Informed by the behaviour change technique (BCT) taxonomy [50], empirical evidence about effectiveness of BCTs, and the literature on hypothesised links between BCTs and behavioural determinants [51], an initial list of BCTs capable of delivering the chosen intervention functions was prepared. The APEASE criteria were then used to refine the selection of the BCTs. Next, the BCTs were operationalised: i.e., each BCT was translated into a feature or application for the purpose of delivering it within an intervention. This list of operationalised BCTs (intervention components) was used as an input for stage three workshops and the first stage four workshop. The final selection of BCTs was informed by HVs’ views of the importance and acceptability of the proposed intervention components in the local context, and what could be practically delivered as a coherent intervention.

#### Findings

We identified an initial list of twenty-five BCTs that are potentially capable of delivering the five chosen intervention functions. Of these, 18 BCTs were assessed as potentially relevant for the intervention. The rationale for selecting them is presented in Additional file 6. The other seven BCTs were assessed as either not suitable in context, or likely to be rejected by HVs. The details of this subjective assessment to exclude the seven BCTs are provided in Additional file 7.

The majority of participants at all stage three workshops rated the proposed 18 BCTs (their operationalised versions) as important and acceptable (summary of findings presented in Additional file 8a and b) except for the BCT ‘Behavioural Practice/Rehearsal’ (BCT 8.1). This BCT which was operationalised as “Role Play”, is considered as an effective BCT for development of skills and enhancing beliefs about capability [51]. However, the majority of participants (all stage three workshops and the first stage four workshop) rated this BCT low for importance and acceptability. The 17 BCTs selected for this intervention target a range of behavioural processes that were identified as relevant from the COM-B analysis of HV-level factors. The links between the HV-level barriers, relevant COM-B domains and intervention functions, and the BCTs (and their operationalised versions) selected for the intervention are summarised in Table 8.

**Table 8:** Details of HV-level barriers (listed in order of Capability, Opportunity, Motivation), intervention functions, selected BCTs and their operationalized versions

HV-level modifiable barriers	COM-B component	Intervention function	BCT label and name	Intervention components: operationalisation of the BCT within the intervention
Lack of knowledge of childhood obesity Lack of familiarity with guidelines Skills (cognitive and interpersonal) for performing the practice behaviours	Psychological capability (Knowledge) Psychological capability (Skills)	Education Persuasion Enablement Training Modelling Enablement	5.1 Information about health consequences 12.5 Adding objects to the environment 4.1 Instruction on how to perform a behaviour 6.1 Demonstration of the behaviour 1.4 Action planning 1.2 Problem-solving 8.7 Graded tasks	Provide information on excess and rapid weight gain in 0-2 year olds; early prevention interventions; present and discuss guidelines Provide HVs with educational materials (training pack) (e.g., copies of slides used in the session, key published papers, links to websites) Provide training pack and information about resources (web-based and key published papers) on best practice techniques Show video clips of good communication with parents on healthy weight; group discussions to include awareness/recognition of best practice and empathic communication techniques HVs discuss what changes they should and can implement in their practice routines and how they will go about it; support HVs to generate their own plans to implement practices they perceive as particularly challenging HVs identify their own barriers to implement recommended clinical behaviours; HVs then work in groups to identify their own solutions to those barriers, which will enable them to perform the clinical behaviours; HVs write down their own ‘if-then’ coping plans to manage barriers Working in groups of 2 or 3, HVs first set easy-to-perform tasks and then proceed to increasingly challenging but achievable tasks until they perform the practice behaviour in a challenging situation
Lack of time/ competing priorities Belief: parents lack interest, motivation, and skills Belief: preventing excess weight gain in young children is parents’ responsibility Belief: Parents perceive heavier infants as healthier Disagreement with evidence underpinning the guidelines Uncertainty about identifying infants as having excess weight Low confidence in successfully performing the behaviours Belief: my advice/ intervention does little to prevent childhood obesity	Physical opportunity Psychological capability (memory, attention) Social opportunity (Social influences); Reflective motivation (Professional role and identity) Reflective motivation (Professional role, Intention) Reflective motivation (Beliefs about capabilities) Reflective motivation (Beliefs about consequences)	Training Enablement Education Persuasion Modelling Persuasion	7.1 Prompts and cues 12.5 Adding objects to the environment 6.3 Information about other’s approval 6.2 Social comparison 9.1 Credible source 12.5 Adding objects to the environment 1.6 Discrepancy between current and expected behaviour 6.1 Demonstration of the behaviour 15.3 Focus on past success 15.1 Verbal persuasion of capability 5.1 Information about health consequences 5.2 Salience of consequences	Prompt HVs to discuss (1) using service delivery prompts as reminders; (2) strategies that can help to reduce time demand and/or competing time demands; Work with HVs to explore potential for designing reminders by adapting existing NHS resources (e.g., ‘Ready to Relate’ cards) [52] Provide HVs with information (UK literature) on parents’ expressed need for support from PCPs and parents’ preferences for how weight related information is communicated; Suggest that raising the topic of child’s weight is particularly important given greater difficulties for parents to initiate the topic because of the social stigma of obesity; suggest that, even if resistance is experienced, discussing the topic will influence the perception of parents (and potentially their practices) Provide information (citing UK and other relevant literature) on (1) positive outcomes of trained (PCP)-led prevention interventions; (2) PCP’s role in motivating parents and correcting misperceptions on healthy weight gain in infants Inform HVs about the credibility of the evidence underpinning the guidelines Provide HVs with educational materials (training pack) Provide information (UK literature) of gaps in evidence-based practices; draw attention to the link between recommended practices and two high impact areas of health visiting (infant nutrition, healthy weight); discuss implications of practice gaps Show video clips of good communication around raising the topic of weight and discussing weight related topics with parents HVs (individually and/in groups of 2-3) reflect on personal experiences of positive and negative weight-related communication in practice; prompt HVs to consider how their existing beliefs impact on their attitudes and intention to perform the behaviours Facilitator provides constructive feedback, links feedback with HV’s ability to provide guidance in real life settings, and counters any doubts with credible arguments Present and discuss motivational videos, testimonials, and success stories (health visiting Case Studies)
Fear of negative reactions from parents Concerns about harm to relationship with parents/ family	Automatic motivation (impulses, habits); Social opportunity (social influences)	Modelling Enablement	6.1 Demonstration of the behaviour 13.2 Framing/reframing 3.2 Social support – practical	Show video clips of sensitive communications with parents that minimise potential offence and embarrassment Reframe discussing weight issues as meeting child/ parent’s needs (focus on child’s health and not on weight); emphasise the role of the ‘obesogenic’ environment Suggest that raising the topic of child’s weight is particularly important given greater difficulties for parents to initiate the topic because of the social stigma of obesity Encourage HVs to use staff meetings to offer their peers and colleagues moral support, positive interaction, sharing and comparison

### Specify form of delivery of the intervention

#### Method

The form of delivery (FoD) of an intervention refers to the way the components of the Intervention are delivered to the recipients of the intervention [53]. The FoD for this intervention was informed by HVs’ views, obtained from group discussions held at stage three and stage four workshops. A plan was developed to “package together” the selected BCTs into a cohesive intervention that could be practically delivered. The delivery mode of a face-to-face interactive workshop was selected because interactive professional development training is familiar to HVs and the evidence from literature [54, 55] suggests that training interventions delivered through interactive workshops have the potential to change practitioners’ behaviours.

#### Findings

The proposed intervention comprises an incentivised (by offering continuing professional development points) interactive face-to-face one-day training session for HVs; a training pack and resources for HVs (e.g., educational materials for HVs and parents, and paper- based practice tools for HVs); a handbook for the facilitator, and awareness raising of the intervention (e.g., posters) amongst staff. An outline of the form of delivery that could be used for the intervention when it is ready for feasibility testing is suggested, using an adapted version of the TIDieR framework (Additional file 9).

**Fig. 2:**
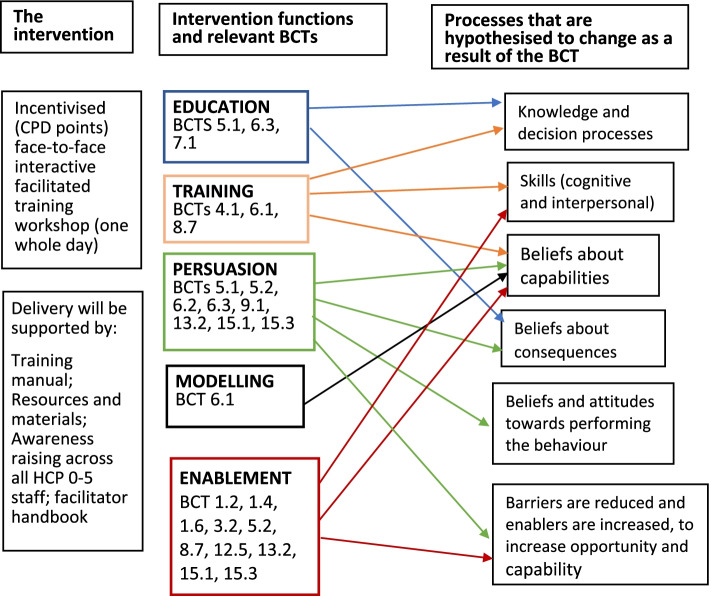
Logic model of the proposed draft intervention: specifying contents and hypothesised mechanisms of change; BCT labels are from BCT taxonomy v1.0

A logic model of the intervention [56] was developed to graphically represent the BCTs included in the proposed intervention and the different hypothesised processes through which they influence behaviour, as shown in Fig. 2.

## Step 4. Identify outcomes and methods for a future feasibility study

### Method

The outcome measures and methods for a future feasibility study were determined in advance, informed by methodological guidance on feasibility studies [57] and HVs’ views of the importance and acceptability of the proposed methods in the local context. Four methodological issues were identified as important areas of focus for a feasibility study of the intervention: recruitment capability, feasibility of delivery (practicality), intervention fidelity, and acceptability of the intervention. The feasibility outcomes related to those four issues and methods that can be used to assess those outcomes are summarised in Table 9.

**Table 9:** Proposed outcomes and methods for a future feasibility study

Area of focus	Proposed outcomes and methods of assessment
Recruitment capability	• Number of service provider organisations who register an interest to participate • Number of service provider organisations declining the offer • Recruitment rate (HVs): number of HVs who were in attendance (expressed as percent of total number of HVs who were invited)
Feasibility of delivery (practicality)	• Time required (number of weeks) for recruitment procedures to be completed • Number of intervention sessions required to deliver the intervention to all recruited HVs at the site • The number of intervention sessions delivered at site with the planned number of HVs (provisionally set as 12 HVs) per session • Time required (in hours) for delivery of each session of the intervention
Fidelity of delivery and fidelity of receipt	• Video-recording of intervention session and intervention facilitator’s completed checklist • 1:1 semi-structured interview with recipients (sub-sample) • 1:1 semi-structured interview with intervention facilitator • Direct observation by trained researcher and researcher’s notes
Acceptability of the intervention to HVs	• Theoretical framework of acceptability questionnaire (7-item, 5 point Likert scale questionnaire) [58]; an open question can be included in the questionnaire for recipients to provide comments • Feedback from recipients (group interview with sub-sample)

### Findings

The proposed methods and measures were considered as relevant and feasible in the local context by majority of stage four workshop participants (Additional file 10).

## Evaluation of the workshops

A total of 123 completed responses were collected from 147 participants who took part in the 11 workshops that covered the stages of the designing of the intervention, with a response rate of 84%. Participants’ evaluation of the workshops indicated that it was possible to meaningfully engage with HVs to inform the intervention development research. The findings showed that, overall, the majority of participants agreed that the information and materials presented at the workshops were easy to comprehend and the techniques and activities used at the workshops facilitated participation and generation of ideas. The survey data was supported by numerous positive observations which suggested that HVs felt that the workshop activities enabled open discussions, reflective thinking, and idea generation:

*“The session was very well planned and identified themes well in current practice and strategies for future health visiting practice”.*

*“Well delivered and very friendly…kept audience engaged and momentum going for delivery”.*

The analysis of open responses indicated that HVs valued the opportunity to take part in the research and that participation had motivated them to reflect upon their current practices. Recognition of HVs’ professional role (and communicating it to them) and their role as collaborators in research that is relevant to their practice was appreciated.

## Discussion

This paper describes the systematic development of a training intervention for HVs that is also conceptualised as a behaviour change intervention. The aim of the intervention is to change HVs’ skills, confidence, intention, and eventually their practice behaviours. The process described here outlines how the theory-driven and evidence-based intervention was developed by involving HVs throughout and integrating their perspectives and preferences. While guided by the BCW, the design process was collaborative and iterative. Not all the steps specified in the BCW framework [19] needed to be implemented. For example, describing the problem of interest and selection of the behaviours of interest for this intervention were pre-specified (selected *‘*a priori*’*) by DCC’s public health department. The BCW guidance suggests identifying appropriate policy-related categories to support the selected intervention functions. This research focused on behaviour change at the level of the individual, and therefore, changing policy was judged as outside the scope of this study. It was assumed that this intervention would fit under the category “service provision”.

The study provides an example of how BCTs can be operationalised and assembled together into a coherent intervention that can be pragmatically delivered. The COM-B analysis of the barriers and facilitators to the behaviours enabled the identification of key behavioural processes to target with an intervention. The proposed links between the COM-B and the TDF facilitated the identification of the specific theoretical domains of behaviour that represented the relevant change processes for this intervention. Targeting specific change processes is recommended, as this may increase the potential for effectiveness of the intervention [59]. The systematic application of the APEASE criteria to contextualise the selection of intervention functions and content (BCTs) increases the potential for acceptability and feasibility of the intervention. However, this process was not straightforward. Subjective decisions that were made in the application of the APEASE criteria were informed by reviewing the health visiting literature and literature on development of BCT-based interventions for health professionals.

The selection of potentially useful BCTs was informed by recent methodological work by which BCTs have been mapped to relevant theoretical constructs of behaviour [51]. However, selecting the most relevant or useful BCTs was challenging. Currently, there is limited understanding of which BCTs or groups of BCTs are likely to be most useful to target a particular theoretical determinant of behaviour. Also, there is little understanding about how different BCTs compare in their effectiveness in inducing behaviour change. Another challenge was translating the BCTs into intervention components. Although the BCT taxonomy provides examples of how each BCT can be operationalised, there is no consensus or guidance in the literature on determining how best to operationalise and deliver selected BCTs within an intervention. The literature suggests that the approach and methods used to operationalise BCTs vary among intervention designers, depending upon the purpose of the intervention and the recipient group [30, 60]. In this study, the operationalisation of the selected BCTs was informed by the existing literature on BCT-based training interventions for practitioners.

### Strengths

A key strength of this study is that it adopted a systematic approach, using both evidence and theory, as recommended by the MRC [18]. Another important strength is the high level of engagement of HVs (as the target recipients of the intervention) across all stages of the designing of the intervention. Our objective was to design an intervention that is rich in context and localised solutions. We recognise that the findings of this research are not necessarily generalisable to other contexts, but they can provide some useful insights to other researchers about what issues may emerge. The co-design approach allowed the integration of evidence synthesised from the literature and evidence generated from the participatory workshops. The strategy for this intervention was determined after conducting a thorough assessment of the appropriate behaviour processes, understanding what it would take to achieve change in those processes, and how best to implement the strategy. The input from HVs was critical to ensure that the design, content, and format of the intervention is acceptable and relevant to HVs and is grounded in HVs’ real-world practice environment (rather than a research environment). This increases the chances that the intervention will be feasible and acceptable to HVs [61].

An important consideration was that the behaviours of interest for this intervention are multiple behaviours. Accounting for both reflective (cognitive) and automatic (impulsivity, habits, emotional processing) motivational processes is strongly recommended for designing of interventions for healthcare professions that aim at multiple behaviour change [62]. The inclusion of planning as a behaviour change strategy to address the post-intentional cognitive processes - action planning and coping planning – has been shown to be effective in adopting a new pattern of behaviour and avoiding previous or undesirable behaviours [63]. As recommended in the literature [62], action planning and coping planning were operationalised into behaviour change techniques and included in this intervention.

Another strength of this research is the use of an established behaviour change taxonomy (BCT taxonomy v1) [50] to select the active ingredients (the BCTs) of the intervention. This ensured that the designing process could draw on a readily available comprehensive list of theory-based BCTs and, that they were defined in a consistent manner throughout the design process (for example, during translating/operationalising the BCTs) and for the documentation of the research. Further, the use of an internationally supported taxonomy to specify intervention content will facilitate (1) faithful delivery of the intervention protocol in practice settings; (2) research efforts to evaluate the effectiveness of the intervention; and (3) the accurate replication of the process by interested researchers [64].

### Limitations

One criticism that can be levelled at this research is that the perspectives of parents of children aged 0-2 years (the service-user or “client” group) were not sought, as additional input, to inform the contents of the intervention. Rather, the barriers and facilitators at the parent/family level were inferred from HVs’ narratives. Evidence suggests that childhood obesity prevention efforts can benefit from an understanding of parental views about infant weight gain [65], the influence of socioeconomic and cultural factors on parents’ infant/child feeding decisions and practices [66], and parents’ preferences about how they want practitioners to engage with them for discussions on weight and weight related behaviours [67]. The decision to include exploratory work with parents was considered but not undertaken after reviewing the scope of this PhD research project and resources (in particular, time) available. Prior to formal feasibility testing, the current version of the intervention will require optimisation (refinement). This will involve testing relevant materials with HVs (for the materials focused for their use) and with parents (for materials to be provided for them), with the aim to improve the quality of the materials and to ensure that the materials are fully understood by the target population [68].

Exploratory work with workshop participants identified contextually relevant determinants of practice and related interventions. It is possible that participants may not have identified all determinants of practice and may have missed determinants that they did not prioritise. Further, the data representing HVs’ perspectives may not have completely revealed the actual cause of their behaviours) but may rather represent attributions HVs made to rationalise their behaviours [69].

This research was undertaken in County Durham where 98% of the population identify themselves as British White/other White. In addition to the socioeconomic environment, cultural and societal factors are important influences on parental beliefs about healthy weight and infant feeding practices, and their efforts to implement practices recommended for healthy child weight [70]. The proposed intervention will need to be adjusted to be culturally competent, before considering the option of adopting the intervention for HVs who work in more ethnically diverse regions in England.

Co-production approaches and the BCW framework are both resource-intensive methods. Involvement of HVs through the different stages of the design process had implications on the use of their time. Using a collaborative approach also presented uncertainties, particularly when workshop participants’ preferences did not align with existing evidence. In the proposed intervention, the BCT ‘behavioural practice/rehearsal’, for which there is evidence [71] that it can help with skills development and induce positive beliefs about capability, was excluded because the workshop data showed that majority of participants rated the BCT (operationalised as “Role Play”) low for importance and acceptability. Challenges of including ‘Role Play’ in skills training have been reported by practitioners and educators [72]. Collaborative approaches to intervention development require deciding how to manage and prioritise different sources of knowledge. However, it is not clear how best to integrate existing evidence from research with stakeholders’ views, particularly when these views are not aligned [35].

The decision-making processes throughout the different stages of the intervention development process required making subjective judgments and pragmatic decisions. It is possible that another researcher would have operationalised selected BCTs in a different way. The literature on intervention development studies often reports using multi-disciplinary consensus meetings and/or workshops to inform decision making throughout the development process [73, 74]. The judgments and decisions taken in this PhD researcher-led study were iteratively reviewed by the researcher’s supervisors (co-authors of this paper) who have extensive experience in development of complex interventions in healthcare.

### Implications for practice and health visiting service provider organisation

Firstly, this study emphasises the need for training and education for HVs (and other practitioner groups) who have a role in prevention of obesity in children. Evidence from the HENRY programme has demonstrated the importance of training for HVs and early years practitioners to enhance their skills and confidence, and work more effectively with parents of pre-school children around excess weight and healthy lifestyle [75]. The need for development of training for practitioners who have a role in prevention of childhood obesity is also recognised in policy statements of the government [76] and the Institute of Health Visiting [77].

Secondly, this research emphasises the importance of a supportive policy and practice environment. In addition to training needs, HVs have identified various resource needs as important facilitators of practices recommended for addressing childhood obesity. The findings from the workshops support existing evidence [49, 78] that translating national guidelines into effective service delivery to prevent childhood obesity will require organisational support for HVs’ role (such as practice tools and clear care pathways) and policies that promote a collaborative approach between different practitioner groups, offer continuity of care, and address HVs’ case workload size.

### Implications for future research

This research has produced the first draft of a new intervention. The next stage of this research is to evaluate the acceptability and feasibility of the intervention in a feasibility study. Prior to formal feasibility testing, this draft version will require further optimisation (refinement). The optimisation process will require working with stakeholders, with the aim to design a prototype version that is ready for feasibility testing. A feasibility study could offer HVs an opportunity to evaluate the content and delivery of the intervention and suggest improvements, to better suit their needs and preferences.

## Conclusions

Prevention of excess weight in children is a challenging public health issue. This paper has described a comprehensive process of developing a face-to-face interactive BCT-based training intervention for HVs to strengthen their role in prevention of excess weight gain in 0-2 year olds in primary care. The intervention was developed by systematic application of theory, collaboration with the target recipients of the intervention, and review of the evidence base. Revisions and adaptations and further refinements will likely be required, informed by input from relevant stakeholders, including the deliverers of the intervention. Subsequently, the intervention should be tested for feasibility and acceptability. The systematic and transparent approach used in the designing of this intervention will facilitate a thorough evaluation via a feasibility study.

## Supplementary Information

**Additional file 1.** Rating of perceived contextual relevance of the SR-identified barriers.

**Additional file 2.** Rating of the perceived contextual relevance of the SR-identified facilitators.

**Additional file 3.** Priority ranking of the barriers.

**Additional file 4.** List of top ten priority ranked barriers.

**Additional file 5.** APEASE criteria rating of the intervention functions that were considered for the study.

**Additional file 6.** Rationale for the selection of the initial list of behaviour change techniques.

**Additional file 7 **Details of the behaviour change techniques that were assessed as *not* practical or likely to be unacceptable by health visitors.

**Additional file 8.** a. Findings of the rating of importance and acceptability of 13 BCTs by workshop participants. b. Findings of the rating of the importance and acceptability of 5 BCTs by workshop participants.

**Additional file 9.** Suggested form of delivery of the intervention informed by the TIDieR framework.

**Additional file 10.** Participants’ rating of proposed outcome measures and methods for a feasibility study of the intervention.

## Data Availability

Data generated or analysed during this study are included in this published article and its supplementary information files.

## Abbreviations

BCT: Behaviour Change Technique
BCW: Behaviour Change Wheel
COM-B framework: Capability, Opportunity, Motivation, Behaviour model
HV: Health visitor
PCP: primary care practitioner
PHE: Public Health England
