# Supplementary material for: A collaborative approach to develop an intervention to strengthen health visitors’ role in prevention of excess weight gain in children
Source: BMC Public Health. 2022 Sep 13;22:1735. doi: 10.1186/s12889-022-14092-x (PMC9469535; doi:10.1186/s12889-022-14092-x)
Supplement: Supplementary file 1 — Additional file 1. Rating of perceived contextual relevance of the SR-identified barriers. [file 12889_2022_14092_MOESM1_ESM.docx]

**Additional file 1. Rating of the barriers**

Note: Rating of SR-identified barriers for their perceived relevance in local context. The barriers that were common to health visitors (HVs) *and* the systematic review (SR), those unique to the SR, and those that were spontaneously mentioned by HVs (but not identified in the SR) are indicated. Emboldening indicates those endorsed by a majority (≥ 50%) of participants.

| Barrier | Description of the barrier (21 were SR-identified) | Rating for relevance by participants (n=29), expressed as % (rounded value) | | | SR-identified barriers | | Barrier not identified in the SR |
| --- | --- | --- | --- | --- | --- | --- | --- |
|  |  | Relevant | Not relevant | Uncertain | Common  to HVs and SR | Unique to the SR |  |
| Practitioner level | Lack of knowledge, skills, and confidence | 42 | 38 | 20 | ✓ |  |  |
|  | Disagreement with guideline(s)/evidence underpinning the  guideline | **52** | 27 | 21 | ✓ |  |  |
|  | Lack of familiarity with guideline content | 49 | 21 | 30 | ✓ |  |  |
|  | Belief: my advice does little to prevent childhood obesity | **53** | 36 | 11 |  | ✓ |  |
|  | Uncertainty about identifying infants as obese | 15 | 66 | 19 |  | ✓ |  |
|  | Belief: prevention primarily a responsibility of parents | **53** | 34 | 13 |  | ✓ |  |
|  | Beliefs about role and responsibilities (uncertainty about own role in prevention) | 26 | 28 | 36 |  | ✓ |  |
| Practitioner- parent  interaction | Fear of offending parents | **60** | 17 | 23 | ✓ |  |  |
|  | Concern about harm to relationship with parents | 30 | 26 | 43 | ✓ |  |  |
| Family level (assumptions and beliefs of practitioners) | Parent/family’s socioeconomic situation | **83** | 7 | 10 | ✓ |  |  |
|  | Lack of motivation/lack of concern | **87** | 6 | 7 | ✓ |  |  |
|  | Lack of knowledge and skills (parenting) | **58** | 16 | 26 | ✓ |  |  |
|  | Perception: heavier infants are healthier | **81** | 11 | 8 | ✓ |  |  |
|  | Parental overweight and lifestyle | **92** | 4 | 4 | ✓ |  |  |
|  | Parents have numerous complex life issues to deal with | **75** | 13 | 11 | ✓ |  |  |
|  | Unhealthy infant/child feeding practices | **85** | 15 | 0 |  | ✓ |  |
|  | Availability of infant foods labelled appropriate for 4 months-old infants in supermarkets | Barrier spontaneously mentioned by participants | | |  |  | ✓ |
| Organisation-level | Time constraints and competing role-related priorities | **79** | 8 | 13 | ✓ |  |  |
|  | Lack of support from organisation (budgets, staffing) | 40 | 30 | 30 | ✓ |  |  |
|  | Lack of obesity training | **60** | 4 | 36 | ✓ |  |  |
|  | Lack of tools and materials for practice | **91** | 4 | 5 | ✓ |  |  |
|  | Lack of collaboration between different practitioner groups | **55** | 10 | 35 | ✓ |  |  |
|  | Regular monitoring of weight in children aged 1-4 years is  not a key performance indicator | Barrier spontaneously mentioned by participants | | |  |  |  |
