# Supplementary material for: A collaborative approach to develop an intervention to strengthen health visitors’ role in prevention of excess weight gain in children
Source: BMC Public Health. 2022 Sep 13;22:1735. doi: 10.1186/s12889-022-14092-x (PMC9469535; doi:10.1186/s12889-022-14092-x)
Supplement: Supplementary file 2 — Additional file 2. Rating of the perceived contextual relevance of the SR-identified facilitators. [file 12889_2022_14092_MOESM2_ESM.docx]

**Additional file 2.** Rating of the facilitators.

Rating of SR-identified facilitators for their perceived relevance in local context; the facilitators that were common to HVs and the SR and those unique to the SR are indicated. Emboldening indicates those endorsed by a majority (≥ 50%) of participants.

| Facilitator | Description of the SR- identified facilitator (n= 10) | Rating for relevance by participants (n=53) expressed as % (rounded value) | | | SR-identified facilitator | |
| --- | --- | --- | --- | --- | --- | --- |
|  |  | Relevant | Not relevant | Uncertain | Common to HVs  and SR | Unique to SR |
| Practitioner level | Familiarity with guideline  content | **68** | 9 | 23 | ✓ |  |
|  | High level of competence  and confidence (self-rated) | **58** | 15 | 26 |  | ✓ |
|  | Ability to use innovative  communication strategies | **70** | 11 | 19 |  | ✓ |
|  | Belief: my advice and  support make a difference | **60** | 25 | 15 |  | ✓ |
| Practitioner- parent interaction | Receptive, engaged  parents | 45 | 25 | 30 | ✓ |  |
|  | Positive relationship with  family | **77** | 4 | 19 | ✓ |  |
| Organisation | Accessible, adequate  training opportunities | 34 | 34 | 32 |  | ✓ |
|  | Availability of practice tools | 47 | 32 | 21 | ✓ |  |
|  | Collaboration between different practitioner groups | 38 | 26 | 36 | ✓ |  |
|  | Support from organisation  for practitioner’s role | **53** | 26 | 21 | ✓ |  |
