# Supplementary material for: A collaborative approach to develop an intervention to strengthen health visitors’ role in prevention of excess weight gain in children
Source: BMC Public Health. 2022 Sep 13;22:1735. doi: 10.1186/s12889-022-14092-x (PMC9469535; doi:10.1186/s12889-022-14092-x)
Supplement: Supplementary file 3 — Additional file 3. Priority ranking of the barriers. [file 12889_2022_14092_MOESM3_ESM.docx]

**Additional file 3**. Ranking of the barriers according to their perceived importance and changeability. Shading shows the top ten priority ranked barriers; *where two barriers had the same score and were therefore had the same rank, the next rank was skipped.

| Level of the barrier | Barrier ID** | # of HVs who rated the  barrier (n=22) | | # of HVs who rated the  barrier (n=22) | | Priority rank score (R) R=I x C | Barrier’s priority rank* |
| --- | --- | --- | --- | --- | --- | --- | --- |
|  |  | Less important | More important(I) | Less changeable | More changeable(C) |  |  |
| Practitioner (HV) | P1 | 3 | 19 | 2 | 20 | 380 | 2 |
|  | P2 | 5 | 17 | 3 | 19 | 323 | 4 |
|  | P3 | 1 | 21 | 3 | 19 | 399 | 1 |
|  | P4 | 4 | 18 | 9 | 13 | 234 | 18 |
|  | P5 | 7 | 15 | 0 | 22 | 330 | 3 |
|  | P6 | 2 | 20 | 9 | 13 | 260 | 11 |
| HV-parent  interaction | P7 | 8 | 14 | 4 | 18 | 252 | 12 |
|  | P8 | 4 | 18 | 7 | 15 | 270 | 9 |
| Parent/ family | F1 | 0 | 22 | 10 | 12 | 264 | 10 |
|  | F2 | 0 | 22 | 11 | 11 | 242 | 16 |
|  | F3 | 3 | 19 | 6 | 16 | 304 | 7 |
|  | F4 | 1 | 21 | 12 | 10 | 210 | 19 |
|  | F5 | 1 | 21 | 7 | 15 | 315 | 6 |
|  | F6 | 2 | 20 | 6 | 16 | 320 | 5 |
|  | F7 | 1 | 21 | 10 | 12 | 252 | 12 |
| Provider organisation | O1 | 1 | 21 | 10 | 12 | 252 | 12 |
|  | O2 | 3 | 19 | 6 | 16 | 304 | 7 |
|  | O3 | 10 | 12 | 5 | 17 | 204 | 20 |
|  | O4 | 4 | 18 | 8 | 14 | 252 | 12 |
|  | O5 | 6 | 16 | 7 | 15 | 240 | 16 |

** Barrier IDs as shown in Table 6 in the main document.
