# Supplementary material for: A collaborative approach to develop an intervention to strengthen health visitors’ role in prevention of excess weight gain in children
Source: BMC Public Health. 2022 Sep 13;22:1735. doi: 10.1186/s12889-022-14092-x (PMC9469535; doi:10.1186/s12889-022-14092-x)
Supplement: Supplementary file 4 — Additional file 4. List of top ten priority ranked barriers. [file 12889_2022_14092_MOESM4_ESM.docx]

**Additional file 4.** The top 10 priority ranked barriers; the ranking of the barriers represented their importance and amenability to change, as perceived by workshop participants.

| Rank | Description of the barrier | Comments |
| --- | --- | --- |
| 1 | Practitioners’ disagreement with guideline/evidence underpinning the guideline(s) | These top 4 priority ranked barriers – all practitioner-level barriers were frequently identified in the systematic review (SR).  In contrast, perceived high level of competence and confidence, and knowledge of guidelines were identified as practitioner-level facilitators within the SR and also by workshop participants. |
| 2 | Practitioners’ lack of knowledge, skills, and confidence |  |
| 3 | Practitioners’ uncertainty about identifying infants as overweight/ obese |  |
| 4 | Practitioners’ lack of familiarity with guideline content |  |
| 5 | Parental overweight and lifestyle | These family/ parent level barriers were frequently identified in the SR and by workshop participants;  participants overwhelmingly ranked these barriers as “important”; however, fewer viewed them as  “changeable”. |
| 6 | Parental lack of knowledge and skills |  |
| 7 | Families with complex health and social issues |  |
| 8 | Lack of tools and resources for practitioners | Most participants ranked this organisational-level barrier as “important”, although fewer ranked it as “changeable”; availability of tools and resources was identified as an important facilitator in the SR and by workshop participants. |
| 9 | Practitioners’ fear of offending parents | Most participants ranked this barrier as “important”; fewer ranked it as “changeable”; the barrier was frequently identified as an important barrier within the SR. Maintaining good relationships with parents was identified as an important facilitator within the SR and also by workshop participants. |
| 10 | Socioeconomic challenges for parents to implement recommended guidance | All participants ranked this barrier as “important”; however, only just over half of all participants considered it as “changeable; this family-level barrier was frequently identified as an important barrier within the SR and by workshop participants. |
