# Supplementary material for: A collaborative approach to develop an intervention to strengthen health visitors’ role in prevention of excess weight gain in children
Source: BMC Public Health. 2022 Sep 13;22:1735. doi: 10.1186/s12889-022-14092-x (PMC9469535; doi:10.1186/s12889-022-14092-x)
Supplement: Supplementary file 5 — Additional file 5. APEASE criteria rating of the intervention functions that were considered for the study. [file 12889_2022_14092_MOESM5_ESM.docx]

**Additional file 5.** APEASE criteria rating of the intervention functions that were considered for the study.

[Abbreviations: HCP 0-5= Healthy Child programme 0-5; HV= health visitor; BCT= behaviour change technique]

| Intervention  function | Affordability | Practicability | Effectiveness & cost- effectiveness effectiveness | Acceptability | Side effects/safety | Equity | Comments | Include  Yes/No |
| --- | --- | --- | --- | --- | --- | --- | --- | --- |
| Education: imparting knowledge and understanding | ✓ | Yes, if limited to 1-day training  training | ? | ✓ | ✓ | ✓ | Affordability: provision of education that would be free to access for health visitors (HVs) has been informally supported by the organisation which is a co-sponsor of this research and commissions the delivery of the HV-led HCP 0-5 service at the research site.  Practicability: information from the workshops and informal discussions with HCP 0-5 managerial staff indicated that providing education would be feasible provided that the intervention will not be too time consuming or burdensome to attend.  Effectiveness and cost-effectiveness: these are not part of a feasibility study but can be evaluated if a pilot trial is planned in the future; educational interventions are more likely to be effective when factors related to the social context and the provider organisations are addressed.  Acceptability: HVs and their managers have welcomed an education intervention as long as it will not be too burdensome or too time consuming for HVs to attend.  Side effects/safety: the risk of undesirable side-effects from providing education that will be based on evidence based guidelines and recommendations has been judged to be minimal.  Equity: providing education that will be based on existing recommendations is not likely to substantially increase disparities in existing healthcare provision/health standards. | Yes |
| Training: imparting skills | ✓ | Same as for Education  day’s training | ? | ✓ | ✓ | ✓ | Similar to education, implementation of skills into routine practice will require organisational support. Delivery of training in skills related to certain practice behaviours (e.g., assessment and monitoring of weight, diet, nutrition, physical and sedentary activities, motivation to change) may be limited due to lack of standardised tools (currently, there is lack of such tools).  The comments made above regarding affordability, practicability, effectiveness and cost-effectiveness, acceptability (with the exception of Rehearsal of the behaviour, a frequently used BCT for this function), side-effects/safety and equity of Education also apply for Training. | Yes |
| Modelling | ✓ | ✓ | ✓ | ✓ | ✓ | ✓ | Demonstration of best practice (indirectly by showing video clips) and directly by trainer and/or an experienced HV from the team (who is considered as a local opinion leader) enacting key practice behaviours is acceptable to HVs. Demonstration of the behaviour is considered as an effective strategy for improving psychological capability([1](#_ENREF_1)). This function was assessed as having met all the criteria. | Yes |
| Environmental restructuring | 🗶 | 🗶 | ? | 🗶 | ? | ? | HVs have highlighted lack of time and practice tools (these barriers linked to the domain “physical opportunity”); these barriers were also a frequent finding in the SR. In contrast, availability of time and practice tools were identified as facilitators in the SR. Environmental restructuring can positively influence (i) physical opportunity (e.g., introducing more mandated visits, allowing HVs more time for consultations, addressing HVs’ caseloads by managing staffing issues, providing time-saving practice tools, providing prompts and reminders); and (ii) social opportunity (e.g., introducing new national-level recommendations for HVs and other PCPs to identify overweight in 0-2 year olds; introducing care pathways, to enable a nationally consistent coherent approach).  The findings from the workshops and the SR strongly suggest that targeting organisational-level barriers is key to support health professionals’ role in prevention of childhood obesity. These findings are also reported in the wider literature([2-4](#_ENREF_2)). However, any restructuring of the practice environment (e.g., additional HV mandated visits, more staff support, better equipment, new guidelines mandating identification of overweight in 0-2 year olds) would require budgetary resources and policy change (at both local and national levels) that is beyond the scope of this intervention. Beyond provision of a training manual (and paper-based educational materials for HVs and parents, as suggested by HVs), restructuring was deemed to be unaffordable and impractical. | No |
| Incentivisation | 🗶 | 🗶 | ? | 🗶 | ? | 🗶 | Incentivisation (financial) as a function for behaviour change was deemed impracticable and not acceptable, in view of the sensitive nature of the topic. Some studies have shown that financial incentives may be effective in changing practices of General Practitioners. The effectiveness and cost-effectiveness of financial incentivisation for increasing compliance with health promotion and prevention care practice (e.g., health visiting practice) is unclear([5](#_ENREF_5)). Currently, financial incentivisation to promote preventive care services in the context of UK primary care does not meet any of the criteria.  Non-financial incentive (**not** as a function for behaviour change) in the form of CPD credit will be provided, to promote participation in the training intervention. | No |
| Restriction | 🗶 | 🗶 | 🗶 | 🗶 | 🗶 | 🗶 | There are already various protocols and guidelines in place. All HVs are registered members of the Nursing and Midwifery Council and are expected to abide by the professional code of conduct of the Council and also the policy regulations of the NHS Trust who employ them. Any new rules or regulations will require change at policy level (locally, if not nationally). Also, HVs’ consider their professional autonomy as an important facilitator of their role. Restriction was considered inappropriate. | No |
| Enablement | ✓ | ✓ | ✓ | ✓ | ✓ | ✓ | Enablement to improve capacity and opportunity to perform the practice behaviours are particularly relevant for this intervention. The intervention is addressing multiple behaviours in practice which are performed alongside several priority competing goal directed behaviours. HVs have expressed keen interest to explore and learn methods that can increase their capacity and opportunity to perform the behaviours. Unlike the Education and Training functions, the effectiveness of this function in actually improving compliance with guidelines is uncertain but this can be tested at a full trial of the intervention. Enablement interventions are likely to be effective when there is organisational support for practitioners’ role. |  |
| Persuasion: | ✓ | ✓ | ? | ? | ✓ | ✓ | Persuasion (persuading HVs to perform the practice behaviours) emerged as a vital intervention function for this intervention, following analysis of individual HV-level barriers. However, HVs already agree that early life interventions are important to address childhood obesity. Yet, efforts to further persuade them to implement guideline-recommended practices are required, in view of the current evidence-practice gap in this field and stubbornly high proportion of children who are already overweight or obese at school age entry. It was decided to include this intervention function as it meets the APEASE criteria (except effectiveness which can be tested at a full trial). | Yes |
| Coercion | 🗶 | 🗶 | 🗶 | 🗶 | 🗶 | 🗶 | HVs enjoy a high level of professional autonomy and take pride in the principles of health visiting practices which serve as the foundations of their preventive care. Creating an expectation of punishment will be unacceptable to HVs, either to participate in the intervention or practice implementation. Any form of coercion will lead to non-engagement with the intervention. | No |

References (relevant only to Additional files section)

1. Connell LE, Carey RN, De Bruin M, Rothman AJ, Johnston M, Kelly MP, et al. Links between behavior change techniques and mechanisms of action: An expert consensus study. Annals of Behavioral Medicine. 2019;53(8):708-20.

2. Mâsse LC, Carbert NS, Scarr J, O'Donnell M. Constraints to implementing guidelines for the identification, assessment, and management of childhood obesity in the clinical care setting: Prevention and treatment framework. Preventive Medicine Reports. 2018;12:87-93.

3. Rudolf M. Observational analysis of disparities in obesity in children in the UK: Has Leeds bucked the trend? Pediatric Obesity. 2018;14(9):e12529.

4. Laws R, Campbell KJ, Pligt P, Ball K, Lynch J, Russell G, et al. Obesity prevention in early life: an opportunity to better support the role of Maternal and Child Health Nurses in Australia. BMC Nursing. 2015;14(1):1-14.

5. Kane R, Johnson P, Town R, Butler M. Economic incentives for preventive care: Summary. AHRQ Evidence Report Summaries: Agency for Healthcare Research and Quality (US); 2004.
