# Supplementary material for: A collaborative approach to develop an intervention to strengthen health visitors’ role in prevention of excess weight gain in children
Source: BMC Public Health. 2022 Sep 13;22:1735. doi: 10.1186/s12889-022-14092-x (PMC9469535; doi:10.1186/s12889-022-14092-x)
Supplement: Supplementary file 6 — Additional file 6. Rationale for the selection of the initial list of behaviour change techniques. [file 12889_2022_14092_MOESM6_ESM.docx]

**Additional file 6.** Rationale for the selection of the initial list of behaviour change techniques (BCTs)

| BCT (label); intervention function | Reasons for choosing the BCT |
| --- | --- |
| Problem solving (1.2)  (Enablement) | The findings from the systematic review (SR) and from the workshop indicated that health visitors (HVs) believe that discussing a child’s weight and other weight related behaviours is challenging (sensitive issue, obesity stigma), particularly with parents who themselves are overweight or there are other social and cultural factors that makes it more difficult. Working with HVs to identify potential problems related to issues that are frequently encountered in real-world practice settings and then identify solutions to overcome those barriers, has the potential to induce positive beliefs about their capability to successfully perform the behaviour. This BCT was considered after taking into consideration the substantial evidence in the published literature, supported by experts’ opinion([1](#_ENREF_1)) for the potential of this BCT to facilitate behaviour change. |
| Action Planning (1.4); (Enablement) | The findings of the SR and data from the workshops indicated that practitioners perceive that performing some of the recommended behaviours as challenging. It is relevant to enable HVs to feel a sense of control of how they will perform the recommended practice behaviours (especially those they find more challenging to implement). Detailed planning of the performance of the behaviour (in particular, those they consider as more challenging to perform) at the training session can enable HVs to feel a sense of control over how they will perform the behaviour in the practice setting. Action planning skills have been shown to help nurses improve their clinical practice performance([3](#_ENREF_6)). Evidence published in behaviour change literature([4](#_ENREF_7)) and opinion of experts ([1](#_ENREF_1)) suggest that this BCT facilitates behaviour change by triggering of the behaviour. |
| Discrepancy between current behaviour and expected practice (1.6)  (Persuasion, Enablement) | The SR found clear evidence that many practitioners (including GPs and health visitors in UK) do not consistently implement the recommended practices, with wide variation among individual practitioners in implementation of the relevant practices. Therefore, it was deemed relevant to draw HVs’ attention to the published evidence in the context of their key role in meeting two high impact public health outcomes: breastfeeding and healthy weight, healthy nutrition. Although there is very limited evidence of the effectiveness of this BCT in published literature, experts believe that drawing attention of the individual between current behaviour pattern and expected behaviours can facilitate behaviour change([1](#_ENREF_1)). |
| Social support (practical) (3.2);  (Enablement) | HVs have described the importance of opportunities to engage in informal conversations with their peers and colleagues about the challenges associated with their practices related to excess weight prevention in very young children. The provision of social support to the person who is attempting to change behaviours is strongly recommended by experts([1](#_ENREF_1)). |
| Instructions on how to perform the behaviour (4.1); (Training) | Findings of the SR and from the workshops have identified the lack of training as an important barrier. HVs have emphasised upon the need for training on how to perform the recommended practices in a manner that has positive outcomes for both HV and parent. This BCT has the potential to improve HVs’ knowledge, skills, and beliefs about their capability to perform the recommended tasks, as suggested by the evidence in the published literature([4](#_ENREF_7)) about the role of this BCT in facilitating behaviour change; inclusion of this BCT in a behaviour change intervention is supported also by experts’ opinion([1](#_ENREF_1)) |
| Provide information about health consequences (5.1); (Education) | The findings of the systematic review (SR) and theoretical analysis of the barriers identified at the health visitor (HV) level from Stage 1 workshops clearly showed the importance of providing information to HVs about obesity during early years and its prevention. HVs have explicitly identified a need to gain knowledge and understanding of excess weight prevention in infants. Systematic reviews([5-7](#_ENREF_8)) have highlighted the beneficial impact of obesity training interventions on practitioner’s knowledge, skills, confidence, and practice patterns. |
| Salience of consequences (5.2); (Persuasion) | The findings from the SR and the workshops indicated that some practitioners are not convinced that obesity prevention must start in infancy and are not aware of the risks of inaction and delay in intervention. Many practitioners believe it is appropriate to discuss overweight prevention only after age of 2 and half years. It is relevant, therefore, to inform HVs about the consequences of delay and also the potential successful outcomes of early intervention. Experts believe this BCT has the potential to induce behaviour change, by changing the person’s beliefs about consequences of performing (or not performing) the recommended behaviour([1](#_ENREF_1)). |
| Demonstration of the behaviour (6.1); (Training, Modelling) | The findings of the SR and data from the workshops clearly indicated that practitioners perceive that performing some of the recommended behaviours (e.g., sensitively raising the topic of weight of an overweight parent who appears to lack interest and not engaged) as challenging. At the workshops, HVs have expressed that they are keen to see experts perform some of the key practice behaviours (e.g., live demonstration by intervention facilitator, or video clips), for the purpose of their own learning and skills development. The published literature([4](#_ENREF_7)), supported by opinion of experts([1](#_ENREF_1)) indicate that this BCT has the potential to induce behaviour change by creating positive beliefs about capability in the practitioner. |
| Social comparison (6.2); (Persuasion) | The findings of the SR and data from workshops indicated that many HVs believe that their prevention efforts are not effective in producing the desired impact on children and families. They believe that their time and resource limited brief consultations are likely to not succeed, given the effects of the ‘obesogenic’ environment of the modern world. Providing information on positive outcomes of trained nurse-led interventions (the evidence for this is rapidly growing) has the potential to induce changes in HV’s existing beliefs about the outcomes of the recommended practices and shape positive attitudes toward performing them. The evidence in the published literature([4](#_ENREF_7)) and experts’ opinion([1](#_ENREF_1)) suggest that this BCT has good potential in facilitating HV behaviour change. |
| Information about others’ approval (6.3); (Persuasion) | The findings of the SR revealed that some practitioners hold the view that (1) parents do not want to, or are not interested, in discussing their child’s weight and weight related behaviours; and (2) parents get upset when they raise the topic of the child’s weight (this is because of the stigma associated with obesity). The SR also identified that a practitioner’s beliefs about what their peers or members of another practitioner group believe and/or implement can influence their practices (for e.g., promoting breastfeeding, discussing overweight in an infant). These views were also expressed by HVs at the workshops. This BCT is believed to facilitate behaviour change by acting on social influences, norms, and subjective norms, based on the evidence from a literature synthesis study([4](#_ENREF_7)) and an expert consensus study ([1](#_ENREF_1)) |
| Prompts, cues (7.1): discuss the role and use of prompts; (Enablement) | Prompts and cues are believed to facilitate behaviour change by aiding practitioner’s memory, attention and decision making processes. The findings of the SR indicated that decision making tools can be particularly helpful for HVs who are required to manage multiple competing role-specific behaviours in time constrained environments. HVs at Stage 1 and 2 workshops have indicated that decision making tools can act as facilitators of HV’s practice behaviours. Evidence from a literature synthesis study([4](#_ENREF_7)) and an expert consensus study([1](#_ENREF_1)) support the rationale of including this BCT. |
| Behavioural practice/ rehearsal (8.1);  (Training) | Rehearsal of the behaviour is widely used as a component in skills training workshops and in behaviour change interventions. There is a substantial evidence base([4](#_ENREF_7)) supported by strong consensus among experts([1](#_ENREF_1)) which endorses the view that this BCT can help in improving skills and facilitate behaviour change by inducing positive beliefs in the individual about their capability to successfully perform the behaviour. HVs have expressed keen interest in developing skills related to raising the topic of weight in a sensitive manner and providing advice and support. The SR too found strong evidence that skills development is an important training need for practitioners. |
| Graded tasks (8.7): (Training, Enablement) | The findings from the SR and from the workshop strongly indicated that practitioners believe that discussing a child’s weight and other weight related behaviours is challenging (sensitive issue, obesity stigma), particularly with parents who themselves are overweight or there are other social and cultural factors that makes it more difficult. Prompting HVs to set easy to perform tasks, making them increasingly difficult, but achievable until they have performed the behaviour can be useful in an educational setting, and can potentially induce positive beliefs about their capability to successfully perform the behaviour. However, in the reality of the practice setting, it is unrealistic to expect that HVs will be able to take a staggered approach to performing the recommended practice behaviours. The evidence from the published literature([4](#_ENREF_7)) and expert opinion([1](#_ENREF_1)) strongly suggests that this BCT can induce positive beliefs about capability. |
| Credible source (9.1); (Persuasion) | At the workshops, HVs have emphasised that the training materials used in the intervention are credible and the intervention facilitator (trainer) must have the appropriate credentials. Experts believe that, to shape an individual’s general beliefs and attitudes about a particular behaviour (with the purpose of persuading the individual to perform the behaviour), the information that is provided to them must originate from a credible source([1](#_ENREF_1)). |
| Adding objects to the environment (12.5); (Enablement) | At the workshops, HVs have expressed the need for practice tools and aids to support implementation of the recommended practices. They also stated that they are keen to receive – as part of the intervention - a training pack and some resources (e.g., educational materials and paper- based practice tools for HVs, and educational materials for parents). Availability of relevant practice based tools are helpful for practitioners who have to manage several competing role-specific tasks and time constraints. The SR findings and evidence in the published literature([2](#_ENREF_2)) emphasise the facilitator role of practice tools and resources for practitioners. Experts believe that adding resources can also trigger the desired behaviour([1](#_ENREF_1)). |
| Framing/ reframing (13.2); (Persuasion, Enablement) | The findings of the SR and the workshops indicated that some practitioners are hesitant to raise the topic of weight and weight related behaviours because of the sensitive nature of the topic and the stigma associated with obesity. It is relevant to suggest to HVs that providing advice about excess weight prevention and management is particularly important given greater difficulties for parents (especially parents who are overweight) to initiate the topic. The literature also recommends that framing discussions about promoting healthy weight in the context of promoting overall health of the child is more likely to produce the intended outcomes([8](#_ENREF_11)). According to the published literature([4](#_ENREF_7)) and experts’ opinion([1](#_ENREF_1)), this BCT facilitates behaviour change by changing the person’s attitudes toward performing the behaviour. |
| Verbal persuasion of capability (15.1) (Persuasion, Enablement) | The findings from the SR and the workshops revealed that practitioners lack confidence in raising the topic of weight of the child and discussing feeding practices with parents. Therefore, providing verbal support and reassurance from a credible intervention facilitator/trainer throughout the training session, telling HVs that they can successfully perform the recommended practices is relevant. There is substantial evidence in the published literature([4](#_ENREF_7)) and consensus among experts([1](#_ENREF_1)) that this BCT can potentially facilitate behaviour change by inducing positive beliefs about capability. |
| Focus on past success (15.3): (Enablement) | The SR found that many practitioners feel demotivated because they believe their role has limited impact on children, owing to numerous other barriers at the level of the parent. At the workshops, HVs expressed lack of optimism about their prevention and health promotion work with regard to child healthy weight. There is evidence in the health visiting literature of the positive impact of reflection on practice (Communities of Practice) and focusing on success stories on inducing positive beliefs and attitudes, and improving practice([9](#_ENREF_12)). Experts believe that this BCT is capable of facilitating behaviour change by inducing positive beliefs in the person about their ability to perform the behaviour([1](#_ENREF_1)); there is some supporting evidence also in the published literature that it is useful ([4](#_ENREF_7)). |

References (relevant only to Additional files section)

1. Connell LE, Carey RN, De Bruin M, Rothman AJ, Johnston M, Kelly MP, et al. Links between behavior change techniques and mechanisms of action: An expert consensus study. Annals of Behavioral Medicine. 2019;53(8):708-20.

2. Mâsse LC, Carbert NS, Scarr J, O'Donnell M. Constraints to implementing guidelines for the identification, assessment, and management of childhood obesity in the clinical care setting: Prevention and treatment framework. Preventive Medicine Reports. 2018;12:87-93.

3. O'Neal H, Manley K. Action planning: making change happen in clinical practice. Nursing Standard. 2007;21(35):35-9.

4. Carey RN, Connell LE, Johnston M, Rothman AJ, de Bruin M, Kelly MP, et al. Behavior Change Techniques and Their Mechanisms of Action: A Synthesis of Links Described in Published Intervention Literature. Annals of Behavioral Medicine. 2018;53(8):693-707.

5. Jacobson D, Gance‐Cleveland B. A systematic review of primary healthcare provider education and training using the Chronic Care Model for childhood obesity. Obesity Reviews. 2011;12(5):e244-e56.

6. Reading JM, Snell LM, LaRose JG. A systematic review of weight-related communication trainings for physicians. Translational Behavioral Medicine. 2020;10(5):1110-9.

7. Yazdizadeh B, Walker R, Skouteris H, Olander EK, Hill B. Interventions improving health professionals’ practice for addressing patients’ weight management behaviours: systematic review of reviews. Health Promotion International. 2020;36(1):165-77.

8. Chadwick P, Sacher P, Swain C. Talking to families about overweight children. British Journal of School Nursing. 2008;3(6):271-6.

9. Manley K, Greaves J. Implementing & evaluating a community of practice for health visiting. Health Education Kent Surrey and Sussex, Kent Community Healthcare Foundation Trust and Medway Community Health; 2016.
