# Supplementary material for: A collaborative approach to develop an intervention to strengthen health visitors’ role in prevention of excess weight gain in children
Source: BMC Public Health. 2022 Sep 13;22:1735. doi: 10.1186/s12889-022-14092-x (PMC9469535; doi:10.1186/s12889-022-14092-x)
Supplement: Supplementary file 7 — Additional file 7 Details of the behaviour change techniques that were assessed as not practical or likely to be unacceptable by health visitors. [file 12889_2022_14092_MOESM7_ESM.docx]

**Additional file 7.** Details of the behaviour change techniques that were assessed as *not* practical or likely to be unacceptable by health visitors.

| BCT name (label) | Description of the BCT | Reason(s) for assessing BCT as *not* practicable or acceptable as a behaviour change strategy |
| --- | --- | --- |
| Goal setting (behaviour)  (1.1) | Prompt HVs set or agree on a goal to perform the practice behaviour | Having specific goals (e.g., assessing child’s weight and discussing findings with parents during visits) as opposed to preventive care goals that health visitors (HVs) already have for children and families is likely to elicit responses ranging from contentious to unacceptable from HVs. Evidence from HV literature suggests that goals that allow measurement of compliance or comparison of performance between individual HVs are likely to be unacceptable([1](#_ENREF_13), [2](#_ENREF_14)); also, currently, there is no national requirement for HVs to identify 0-2-year-olds who may be overweight([3](#_ENREF_15)). |
| Commitment  (1.9) | Ask HVs to affirm or reaffirm statements indicating commitment to change the behaviour | Not considered practical to ask HVs to commit to a specific task (for e.g., weight and length assessment) at every mandated visit; although some HVs may affirm this, HVs have emphasised they face many competing demands (largely determined by organisational priorities) for their time([4](#_ENREF_16)). Also, currently, there is no national requirement for HVs to identify 0-2-year-olds who are overweight ([3](#_ENREF_15)). HVs are expected to use their professional judgement when deciding whether to record an infant/toddler’s weight; asking HVs to make a commitment to specific tasks may in fact cause a sense of failure if HVs do not or cannot act upon their commitment. |
| Feedback on behaviour (2.2) | A peer (or supervisor) monitors and provides feedback on performance of the practice behaviour | Many relevant behaviours take place at client’s home; resource constraints (staffing and workload pressure) mean that it will not be feasible for peer/supervisor to mutually agree to monitor and provide feedback on individual HV’s practice behaviours. Feedback from parents/carers about experiences of care provided by HVs can provide valuable information about HV-delivered care and could be considered as a data source in a future study to evaluate the effects of the intervention([5](#_ENREF_17)). |
| Self-monitoring  of the behaviour  (2.3) | Establish a method for HVs to monitor their performance of the recommended practice behaviours | HVs already document the tasks they perform using prescribed forms. Self-monitoring of behaviour when combined with goal setting, feedback on behaviour, and monitoring of behaviour have been used with increased intervention effects to support healthy lifestyle behaviours([6](#_ENREF_18), [7](#_ENREF_19)). Nonetheless, in the current policy and practice environment of HVs’ professional work, this BCT was not considered as a practical strategy for professional behaviour change because: (1) currently, there is no national requirement for HVs to identify 0-2-year-olds who are overweight([3](#_ENREF_15)); (2) HVs are recommended to use their professional clinical judgement when making decisions about frequency of weighing infants and intervening. Therefore, this BCT is unlikely to motivate HVs in the absence of an initiative from their employer organisation. Information routinely collected by HVs could be a useful data source in a future study to evaluate the intervention rather than as a behaviour change strategy. |
| Self-monitoring of outcomes of the behaviour (2.4) | Establish a method for HVs to monitor the outcomes of their performance of the recommended practice behaviours | HVs already record the outcomes of the tasks that they perform during their contacts with children and families, as mandated by their employer organisation. This BCT was not considered for inclusion as a behaviour change strategy because of reasons stated above for BCT 2.3; information routinely collected by HVs could be a useful data source in a future study, but to evaluate the intervention rather than as a behaviour change strategy. |
| Feedback on the outcomes of the behaviour (2.7) | A peer (or supervisor) monitors and provides feedback on the  outcome(s) of the behavior | Not considered as practical for reasons stated above for BCT 2.2 |
| Mental rehearsal of successful performance (15.2) | Advise HVs to practise imagining performing the behaviour successfully in relevant contexts | Not considered as practical; the behaviours addressed by this intervention are too complex to meaningfully imagine performing them |

References (relevant only to Additional files section)

1. Greenway JC, Entwistle VA, terMeulen R. Ethical tensions associated with the promotion of public health policy in health visiting: a qualitative investigation of health visitors’ views. Primary Health Care Research & Development. 2013;14(2):200-11.

2. Appleton JV, Cowley S. The guideline contradiction: health visitors’ use of formal guidelines for identifying and assessing families in need. International Journal of Nursing Studies. 2004;41(7):785-97.

3. Hickson T. Evaluation of the use of a Healthy Weight Discussion Tool by a health visiting team. Journal of Health Visiting. 2019;7(6):272-8.

4. Redsell SA, Atkinson PJ, Nathan D, Siriwardena AN, Swift JA, Glazebrook C. Preventing childhood obesity during infancy in UK primary care: a mixed-methods study of HCPs' knowledge, beliefs and practice. BMC Family Practice. 2011;12(1):1-9.

5. Donetto S, Malone M, Hughes J, Morrow E, Cowley S, Maben J. Health visiting: the voice of service users2013. Available from: https://www.kcl.ac.uk/nmpc/research/nnru/publications/reports/voice-of-service-user-report-july-2013-final.pdf.

6. Michie S, Fixsen D, Grimshaw JM, Eccles MP. Specifying and reporting complex behaviour change interventions: the need for a scientific method. Implement Science. 2009;4:40.

7. Dombrowski SU, Sniehotta FF, Avenell A, Johnston M, MacLennan G, Araújo-Soares V. Identifying active ingredients in complex behavioural interventions for obese adults with obesity-related co-morbidities or additional risk factors for co-morbidities: a systematic review. Health Psychology Review. 2012;6(1):7-32.
