# Supplementary material for: A collaborative approach to develop an intervention to strengthen health visitors’ role in prevention of excess weight gain in children
Source: BMC Public Health. 2022 Sep 13;22:1735. doi: 10.1186/s12889-022-14092-x (PMC9469535; doi:10.1186/s12889-022-14092-x)
Supplement: Supplementary file 8 — Additional file 8. a. Findings of the rating of importance and acceptability of 13 BCTs by workshop participants. b. Findings of the rating of the importance and acceptability of 5 BCTs by workshop participants. [file 12889_2022_14092_MOESM8_ESM.docx]

**Additional file 8a.** Findings of the rating of importance and acceptability of **13** BCTs by workshop participants (**n=26**).

| Name of the BCT (BCT label); | % (rounded value) of HVs  (n=26) who rated the BCT | | | | Decision to select BCT (based on HVs’ input and APEASE criteria) |
| --- | --- | --- | --- | --- | --- |
|  | Importance | | Acceptability | |  |
|  | Low | High | Low | High |  |
| Discrepancy between current behaviour and expected practice (1.6) | 8 | 92 | 8 | 92 | **Yes,** HVs are keen to know about gaps in evidence- based practice |
| Social support (practical) (3.2): use opportunities to share experiences and provide practical help (from colleagues) | 8 | 92 | 8 | 92 | **Yes**, HVs suggested routine monthly staff meetings could be used to facilitate peer social support |
| Instructions on how to perform the behaviour (4.1); | 4 | 96 | 4 | 96 | **Yes**, HVs value skills training |
| Provide information about health consequences (5.1) | 0 | 100 | 0 | 100 | **Yes**, HVs want updated information on  early childhood obesity |
| Salience of consequences (5.2): emphasise the consequences of intervening early (and delay in intervention) | 8 | 92 | 12 | 88 | **Yes,** HVs are keen to explore and learn about the benefits of early prevention |
| Social comparison (6.2): provide information on positive outcomes of trained nurse-led prevention interventions | 4 | 96 | 8 | 92 | **Yes**, HVs want to know more about the role of practitioners in prevention of excess weight gain during early years |
| Information about others’ approval (6.3): provide information about what other people think about the behaviour | 0 | 100 | 0 | 100 | **Yes**, HVs want to know about families’ expressed need for care and their experiences of receiving preventive care |
| Prompts, cues (7.1): discuss the role and use of self-designed prompts | 15 | 85 | 15 | 85 | **Yes**, HVs believe prompts are helpful and have expressed support to discuss their use |
| Credible source (9.1): present information from a credible source | 1 | 100 | 0 | 100 | **Yes**, this feature is highly desirable by all HVs |

**Additional file 8b**. Findings of the rating of importance and acceptability of **5** BCTs by workshop participants (**n= 46**).

| Name of the BCT (BCT label) | % (rounded value) of HVs  (n=46) who rated the BCT | | | | Decision to select BCT (based on HVs’ input and APEASE criteria) |
| --- | --- | --- | --- | --- | --- |
|  | Importance | | Acceptability | |  |
|  | Low | High | Low | High |  |
| Problem solving (1.2): analyse factors that help/hinder the performance of the behaviour and then devise solutions | 22 | 78 | 11 | 89 | **Ye**s, there is support from HVs for activities that can help with skills development |
| Action Planning (1.4): plan how they will go about performing behaviours they consider are difficult/complex to perform | 11 | 89 | 6 | 94 | **Yes**, HVs are keen to learn about how to plan in advance practice behaviours they find challenging to implement |
| Demonstration of the behaviour (6.1): provide sample of the performance of the behaviour | 26 | 74 | 13 | 87 | **Yes**, HVs want to see examples of best practice |
| Behavioural practice/rehearsal (8.1): practice performance of the behaviour | 59 | 41 | 83 | 17 | **No,** majority of HVs did not accept this BCT (operationalised as Role  Play) |
| Graded tasks (8.7):  Set with easy- to-perform tasks, making them increasingly difficult, but achievable, until behaviour is performed | 37 | 63 | 35 | 65 | **Yes,** there is support from HVs for this BCT linked to skills development |
