# Supplementary material for: A collaborative approach to develop an intervention to strengthen health visitors’ role in prevention of excess weight gain in children
Source: BMC Public Health. 2022 Sep 13;22:1735. doi: 10.1186/s12889-022-14092-x (PMC9469535; doi:10.1186/s12889-022-14092-x)
Supplement: Supplementary file 9 — Additional file 9. Suggested form of delivery of the intervention informed by the TIDieR framework. [file 12889_2022_14092_MOESM9_ESM.docx]

**Additional file 9.** Suggested form of delivery of the intervention informed by the TIDieR framework

| Delivery elements and  features | Description |
| --- | --- |
| Provider (who delivers and facilitates the intervention?)  [TIDieR: Who] | |
| Professional background | Preferred: Health visiting; Infant nutrition |
| Professional experience | To be confirmed |
| Number of providers | To be confirmed |
| Delivery format (what are the methods of delivering the training intervention?)  [TIDieR: How] | |
| Mode of delivery | Face to face facilitated interactive training workshop for small groups of health visitors (HVs) (suggested 12 HVs per session) |
| Materials (what materials are being used to deliver the intervention content?  [TIDieR: What?] | |
| Pre- workshop reading material (could be made available online):  At the workshop: Training pack (containing all teaching materials and a workbook), guidebook for families, PowerPoint slides, videos of examples of good practice, case stories (health visiting communities of practice) | |
| Procedures (what procedures will be used to deliver the intervention?) [TIDieR: What?] | |
| Mix of didactic lectures and interactive skills development activities; presentations aided by PowerPoint slides; video clips; self-reflection on practice; case stories (health visiting community of practice); individual and group based activities (e.g., context-bound communication training skills, action planning, coping planning, graded tasks); discussion and feedback from peers and facilitator(s); provide certificates of attendance to participants | |
| Setting (Where is the intervention being delivered?) [TIDieR: WHERE?] | |
| Sessions are delivered separately to each location based HV team; priorities for HVs are: a facility that is ‘local’ (not involve too much travel time), adequately equipped to host an interactive training workshop, and has adequate parking facilities | |
| Intensity (What is the intensity with which the intervention is being delivered?) [TIDieR: When and how much? | |
| HVs have suggested that participation is likely to be higher if intervention sessions are delivered separately to each location based HV team. Each training session will take one full working day (5 hours and 35 minutes with an additional 70 min for breaks). All HVs will have the opportunity to participate once in the training workshop. | |
