# Supplementary material for: A collaborative approach to develop an intervention to strengthen health visitors’ role in prevention of excess weight gain in children
Source: BMC Public Health. 2022 Sep 13;22:1735. doi: 10.1186/s12889-022-14092-x (PMC9469535; doi:10.1186/s12889-022-14092-x)
Supplement: Supplementary file 10 — Additional file 10. Participants’ rating of proposed outcome measures and methods for a feasibility study of the intervention. [file 12889_2022_14092_MOESM10_ESM.docx]

**Additional file 10**. Participants’ rating of proposed outcome measures and methods for a feasibility study of the intervention.

| № | Feasibility outcomes and methods of assessment | % (rounded value)  of HVs (n=26) who rated the method | |
| --- | --- | --- | --- |
|  |  | Important | Feasible |
| 1 | Recruitment rate: number of HVs attending the intervention session expressed as a percentage of HVs who were invited to the session | 77 | 81 |
| 2 | Acceptability of the intervention content: questionnaire pre- and post-intervention (to be completed on the day of the session) | 88 | 100 |
| 3 | Acceptability of the intervention: group interviews with intervention recipients (sub-sample) on day of intervention | 81 | 65 |
| 4 | Feasibility of delivery: Number of sessions required to deliver the intervention to all HVs who have confirmed their intention to participate | 85 | 85 |
| 5 | Feasibility of delivery: Time (in hours) required for delivery of the intervention session at the site | 65 | 77 |
| 6 | Feasibility of delivery: Number of sessions delivered with the planned number of HVs (suggested: 12 per session) in attendance at the session | 86 | 86 |
| 7 | Fidelity of delivery: Audio-video recording of the intervention session by trained independent researcher | 73 | 50 |
| 8 | Fidelity of receipt: interviews with intervention recipients (sub- sample): HVs’ self-reported comprehension of, and engagement with intervention activities | 92 | 92 |
| 9 | Fidelity of receipt: 1:1 interview with *intervention facilitator*  (explore facilitator’s subjective assessment of receipt of  intervention based on their direct observation of recipients’ verbal  understanding and performance of skills) | Not presented to HVs for rating | |
